# Optimized Taste-Masked Microparticles for Orally Disintegrating Tablets as a Promising Dosage Form for Alzheimer’s Disease Patients

**DOI:** 10.3390/pharmaceutics13071046

**Published:** 2021-07-09

**Authors:** Lalinthip Sutthapitaksakul, Kasitpong Thanawuth, Crispin R. Dass, Pornsak Sriamornsak

**Affiliations:** 1 Faculty of Pharmacy, Silpakorn University, Nakhon Pathom 73000, Thailand; Sutthapitak_l@silpakorn.edu (L.S.); Thanawuth_k@silpakorn.edu (K.T.); 2 Pharmaceutical Biopolymer Group (PBiG), Silpakorn University, Nakhon Pathom 73000, Thailand; 3 Faculty of Health Sciences, Curtin Medical School, Curtin University, Perth 6845, Australia; crispin.dass@curtin.edu.au; 4 Curtin Health Innovation Research Institute, Bentley 6102, Australia; 5 Academy of Science, The Royal Society of Thailand, Bangkok 10300, Thailand

**Keywords:** taste masking, microparticles, orally disintegrating tablets, Box-Behnken design, donepezil hydrochloride

## Abstract

The objective of this research was to optimize the tasted-masked microparticles for orally disintegrating tablets containing donepezil hydrochloride using quality risk assessment and design of experiment approaches. The double emulsion solvent evaporation technique using aminoalkyl methacrylate copolymer (AMC) was used to prepare taste-masked microparticles. Factors affecting the quality of the taste-masked microparticles were analyzed using an Ishikawa diagram. A risk-ranking approach was used to rank the formulation and process risks. Furthermore, the effect of AMC quantity, stirring time, and volume of outer water phase on various responses, such as particle size, the amount of drug dissolved at 5 min (Q_5_) in simulated saliva fluid, and mean dissolution time (MDT) in simulated gastric fluid, was investigated using the Box-Behnken design. The optimized microparticles were then used to prepare orally disintegrating tablets (ODTs) and evaluated by in vitro and in vivo testing. The results demonstrated that particle size was influenced by the AMC amount and stirring time. Q_5_ was significantly affected by the amount of AMC and the volume of the outer water phase. On the other hand, these two factors had a positive effect on MDT. The optimized microparticles had a particle size of 174.45 ± 18.19 µm, Q_5_ of 5.04%, and MDT of 5.97 min. The ODTs with taste-masked microparticles showed acceptable in vitro dissolution with an MDT of 5 min. According to the results of a panel of six human volunteers, they greatly improved palatability.

## 1. Introduction

In recent years, the global demographic structure has shifted from younger to older populations, with the proportion of people aged above 65 expected to rise from 703 million to 1.5 billion by 2050 [1]. Among several age-associated health issues, the prevalence of neurodegenerative diseases, such as Alzheimer’s and other types of dementia, is exceptionally high in this population. Alzheimer’s disease affects about 10% of people aged above 65 and 50% of people aged above 85 years, with the number expected to reach 131 million by 2050 [2]. The most promising prescribed medicine in all stages of the disease is donepezil hydrochloride (DPH), which belongs to a class of cholinesterase inhibitors [3,4]. In Alzheimer’s disease patients, 84–93% of those in the moderate to severe stages of the condition had difficulty swallowing [5]. The commercial DPH products for oral administration, such as conventional tablets and capsules, may not be suitable for these individuals [6]. Therefore, the development of DPH orally disintegrating tablets (ODTs) may be a good option because they are designed to rapidly disintegrate into small fragments after being placed on the tongue, facilitating ease of administration in patients who have difficulty swallowing. Since the ODTs are designed to disintegrate in the oral cavity, one of the most important factors to consider in the development of ODTs containing DPH is the use of an effective strategy to mask the severe bitter taste with a numbing sensation. Although numerous strategies could be used to disguise the taste of a drug, the best strategy should be chosen based on the physicochemical properties of DPH. As DPH is a highly water-soluble drug (55 mg/mL, 25 °C), a microencapsulation technique using emulsion solvent evaporation could be useful in its formulation [7]. Despite the fact that the oil-in-water (O/W) emulsification solvent evaporation technique is widely used, it may not be suited for DPH, since it may rapidly diffuse from the inner to outer water phase during preparation. For this reason, water-in-oil-in-water (W/O/W) double emulsion solvent evaporation (DESE) could be a potential approach for encapsulating such drugs [8,9]. This approach has been used to encapsulate small hydrophilic molecules with different purposes, such as propranolol hydrochloride [10], alendronate [11], and clonidine hydrochloride [12]. 

Materials, processes, and environmental factors, such as the polymer amount, volume ratio of dispersed phase to continuous phase, quantity of drug in the dispersed phase, surfactant concentration, agitation factors, evaporation temperature, and others, have been shown to affect microparticle properties in previous studies [8,13]. In this study, the Box-Behnken design (BBD) was used to evaluate the effect of each factor and facilitate the optimization process in order to manufacture taste-masked DPH-loaded microparticles with desirable qualities using the DESE approach. Aminoalkyl methacrylate copolymer (AMC) was chosen as a taste-masking polymer. Following the optimization of the formulation, the microparticles were prepared, characterized, and incorporated into ODTs. The in vitro and in vivo experiments were carried out to evaluate disintegration characteristics and taste-masking performance of ODTs containing DPH-loaded microparticles.

## 2. Materials and Methods

### 2.1. Materials

DPH (lot number 000000085) was kindly supplied by Pharma Nueva Co., Ltd., Bangkok, Thailand. AMC (Eudragit^®^ E PO, lot number G170331544) was kindly provided by Evonik Röhm GmbH, Darmstadt, Germany. PVA (lot number 16796TJV), molecular weight of 85,000 Da to 124,000 Da, and degree of hydrolysis of 87% to 89%, was purchased from Sigma-Aldrich, St. Louis, MO, USA. Mannitol (lot number 302004308) was purchased from Shandong Tianli Pharmaceutical Co., Ltd., Shandong, China. Spray dried lactose monohydrate (Supertab^®^ 11SD, lot number 23034009) was purchased from DMV-Fonterra Excipients GmbH & Co., Goch, Germany. Microcrystalline cellulose (Comprecel^®^ M101D+, lot number C2006037) was purchased from Mingtai Chemical Co., Ltd, Taoyuan City, Taiwan. Crospovidone (Polyplasdone^®^ XL, lot number 0002434812) and polyvinyl pyrrolidone K-30 (PVP K-30; lot number 002377911) were from Ashland Chemical Inc., Wilmington, DE, USA. Magnesium stearate (Kemilub^®^ EM-F-V) was purchased from Italmatch Chemicals, Genova, Spain. All other chemicals and solvents used in this study were of reagent grade or high-performance liquid chromatography grade and were used as received. 

Simulated saliva fluid (SSF) was prepared by dissolving 12 g of Na_2_HPO_4_·7H_2_O and 7.6 g of NaH_2_PO_4_·H_2_O in 800 mL of distilled water, adjusting to pH 6.75 using HCl or NaOH, and then adjusting to 1000 mL with distilled water. Simulated gastric fluid USP without pepsin (SGF, pH 1.2) was prepared by dissolving 3 g of NaCl in 1450 mL of distilled water and then adjusting the pH to 1.2 using diluted HCl.

### 2.2. Identification of Factors Affecting Microparticle Preparation Using Quality Risk Assessment

The quality risk assessment approach was used in this work to aid in the selection of factors affecting microparticle preparation. A fishbone diagram based on previous reports was used to identify the relevant factors influencing the characteristics of drug-loaded microparticles produced by DESE. The risk matrix was subsequently established to prioritize factor risk by multiplying the probability and its impact. The risk value of each factor was then classified as high, medium, and low risk [14,15]. 

### 2.3. Microparticle Preparation by DESE Technique

A two-step emulsification approach was used to produce DPH-loaded double emulsions. To prepare the inner water phase (W_1_), DPH was first dissolved in distilled water (10 mL), and then AMC was dissolved in dichloromethane (25 mL) to prepare the oil phase (O). Then, an ultrasonic processor (UP400S, Hielscher, Teltow, Germany) with 100% amplitude of power (400 W, 24 kHz) equipped with H14 horn was used to mix both phases, for 2 min, to form a primary water-in-oil (W_1_/O) emulsion. The primary emulsion was subsequently emulsified with 1% PVA solution (350–450 mL) as an outer water phase (W_2_) using an overhead stirrer (SS20, Stuart, Staffordshire, UK) at 500 rpm to make (W_1_/O/W_2_) double emulsions. The PVA solution was prepared with carbonate-bicarbonate buffer (pH 10). After that, double emulsions were agitated again using a magnetic stirrer to help remove the organic solvent. The solid microparticles were recovered using an ultracentrifuge machine set at 7000 rpm for 10 min and washed for 3 times with distilled water to remove free DPH and PVA residue. Then, the resultant product was dried under a vacuum drying chamber at 30 °C for 12 h before being kept in a desiccator cabinet for future testing. 

### 2.4. Optimization of DPH-Loaded Microparticles Using BBD

BBD was used as a tool to investigate and optimize the formulation and processing factors. The preliminary study results [16,17] were used to assess the level of each factors. Design-Expert^®^ software (Stat-Ease Inc., Minneapolis, MN, USA), version 8.0.7.1 was used for the experimental design and analysis. A total of 17 experimental runs with center points were created and executed in a random sequence based on the run order. The statistical significance of the model was assessed using a *p*-value of less than 0.05. An insignificant lack-of-fit was used to determine the suitability of the model while the coefficient of determination (R^2^) was used to determine the goodness of fit of the model. The additional experiments were used to test the validity of the model.

The difference between predicted and actual value was determined by root mean square error (RMSE) by Equation (1):

(1) $$\mathrm{RMSE}=\sqrt{\frac{\sum_{i=1}^{n}{\left(p_{i}{-\;a}_{i}\right)}^{2}}{n}}$$

where p_i_ and a_i_ are predicted value and actual value of experiment, respectively, n is number of experiments.

The percentage of prediction error from optimum condition for preparing DPH-loaded microparticles was calculated as follows:

(2) $$\mathrm{Prediction}\;\mathrm{error}=\left|\frac{p_{i}{-\;a}_{i}}{a_{i}}\right|\times 100$$

### 2.5. Characterization of DPH-Loaded Microparticles

#### 2.5.1. Size Determination of Microparticles by Image Analysis

The microparticles were placed onto a clean glass slide and covered with a cover slip before examining under light microscope (CX41RF, Olympus, Tokyo, Japan). A total of 50 microparticles were imaged (10 microparticles/frame, 5 frames). The Feret diameter of each microparticle was determined by an open-source image analysis software, JMicroVision (available at https://www.jmicrovision.com/). Each formulation was measured in triplicate and the particle size was reported as a median and standard deviation.

#### 2.5.2. Morphology of Microparticles

A scanning electron microscope (SEM; MIRA3, Tescan, Brno, Czech Republic) was used to examine the shape and surface morphology of microparticles. Prior to investigation, the microparticles were attached on sample stub by double-sided adhesive tape and then coated with gold in a vacuum chamber. Images were obtained at an accelerating voltage of 5.0 kV.

#### 2.5.3. Drug Content Measurement

The determination of DPH amount in resultant microparticles was carried out using HPLC (Jasco PU2089 plus quaternary gradient inert pump and a Jasco UV-2070 plus multi wavelength UV-VIS detector, Jasco, Tokyo, Japan). The mixture of 0.01 M phosphate buffer, methanol, and acetonitrile (50:30:20 *v*/*v*) was prepared and adjusted to pH 2.7 ± 0.1 by phosphoric acid. After filtration through 0.45-µm nylon membrane filter, it was degassed and used as a mobile phase. The microparticles were placed into 25-mL volumetric flask and dissolved in 10-mL mobile phase using ultrasonic bath for 15 min. The mixture was adjusted to the volume before passing through a 0.45-µm pore size syringe filter. Then, 20 µm of each sample was injected to an HPLC system with flow rate of 1 mL/min and UV detection wavelength of 270 nm. The drug loading and encapsulation efficiency (EE) were calculated using the following equations:

(3) $$\%\;\mathrm{Drug}\;\mathrm{loading}=\frac{\mathrm{Amount}\;\mathrm{of}\;\mathrm{DPH}\;\mathrm{in}\;\mathrm{microparticles}}{\mathrm{Amount}\;\mathrm{of}\;\mathrm{microparticles}}\times 100$$

(4) $$\%\;\mathrm{EE}=\frac{\mathrm{Amount}\;\mathrm{of}\;\mathrm{drug}\;\mathrm{in}\;\mathrm{microparticles}}{\mathrm{Amount}\;\mathrm{of}\;\mathrm{theoretical}\;\mathrm{drug}\;\mathrm{loading}}\times 100$$

#### 2.5.4. Determination of Residual Solvent

The residual dichloromethane in DPH-loaded microparticles was measured using the headspace technique with a TRACE1310 gas chromatograph (GC; Thermo Fisher Scientific, Waltham, MA, USA) equipped with a TriPlus RSH autosampler and a TSQ9000 mass spectrometer (Thermo Fisher Scientific, USA). The sample was placed in a vial and heated before being injected into Rtx-624 column (30 m × 0.32 mm, 1.80 m; Restek Corporation, Bellefonte, PA, USA). The GC oven was initially set at 35 °C for 10 min, then increased to 200 °C for 2 min at a ramp rate of 10 °C/min. 

#### 2.5.5. Fourier Transform Infrared (FTIR) Spectroscopy

To investigate the effect of preparation factors on the drug-polymer interaction, DPH, AMC, PVA, their physical mixture, and optimized microparticles were pulverized with dried KBr pellets and compressed with a hydraulic press machine. Each sample disk was analyzed over a scan range of 4000 to 400 cm^−1^ by FTIR spectrophotometer (Nicolet 4700, Thermo Electron Corporation, Waltham, MA, USA).

#### 2.5.6. Powder X-ray Diffractometry (PXRD)

The PXRD patterns of DPH, AMC, PVA, their physical mixture, and optimized microparticles were obtained using a powder X-ray diffractometer (Miniflex II, Rigaku, Tokyo, Japan). Each sample was packed on a well of sample holder and positioned in the instrument. The PXRD patterns were recorded by graphite monochromatized Cu Kα radiation in 2-theta range of 5 to 50 degrees at 30 kV and 15 mA with a scan speed of 4 degree/min.

#### 2.5.7. Differential Scanning Calorimetry (DSC) Measurement

Thermal behavior of DPH, AMC, PVA, physical mixture, and optimized microparticles was investigated on differential scanning calorimeter (DSC8000, Perkin Elmer, Boston, MA, USA) with indium as a reference. The samples of approximately 2 to 5 mg were placed inside a sample pan, and hermetically sealed with the lid using standard sample pan crimper. After that, the sample and reference pan were heated from 30 to 300 °C with a heating rate of 10 °C/min under an inert nitrogen purge with a flow rate of 20 mL/min. The DSC thermograms were recorded as a function of temperature.

#### 2.5.8. In vitro Dissolution Test of Microparticles

In order to keep sink condition, the microparticles equivalent to 5 mg of DPH were used; the concentration of the DPH was less than 1% of its solubility in the medium. The drug dissolution from microparticles was determined using a closed system flow-through cell (USP apparatus 4; CE7smart, Sotax AG, Zürich, Switzerland) with a CY7 piston pump and C613 fraction collector. A 5-mm diameter ruby bead was positioned on the apex of a cell for powder and granulates, followed by addition of 1-mm glass beads to form a glass bead bed. After that, a screen with a diameter of 0.2 mm and glass microfiber filters with a pore size of 2.7 µm were placed on the glass bead bed. The microparticles were weighed and placed in cell chamber. Then, 0.2-mm diameter application was positioned before placing 0.7-µm and 2.7-µm pore size glass membrane filters on outlet opening. Thereafter, the test cells were inserted into a cell block. After the dissolution medium temperature reached 37.0 ± 0.5 °C, the test was carried out with 50-mL SSF (pH 6.75) at the flow rate of 4 mL/min, under a sink condition. At the predetermined time, 3 mL of the media were withdrawn and replenished with an equal volume of fresh dissolution media. Afterwards, the drug dissolution in SSF was calculated and used to determine the bitterness of DPH. In the same way, drug dissolution testing of microparticles in SGF (pH 1.2) was carried out with this method. However, SGF was used as a dissolution medium instead of SSF. The quantity of DPH in each sample was determined using HPLC. The mean dissolution time (MDT) in SGF was calculated based on the following equation:

(5) $$\mathrm{MDT}=\frac{\sum_{j=1}^{n}\hat{t_{j}}\Delta M_{j}}{\sum_{j=1}^{n}\Delta M_{j}}$$

where j is number of samples, n is the number of dissolution sample times, $\hat{t_{j}}$ is the time at the midpoint between t_j_ and t_j−1_, which can be calculated from $\hat{t_{j}}$ = (t_j_ + t_j−1_)/2, and $\Delta M_{j}$ is the additional amount of drug dissolved between t_j_ and t_j−1_.

### 2.6. Preparation of ODTs

The ODTs containing optimized microparticles (referred to as OM-ODTs) were prepared and compared with DPH-loaded ODTs (referred to as DPH-ODTs). Table 1 shows the formulation of ODTs containing DPH and optimized microparticles. The direct compression method was used to manufacture tablets with a total weight of 200 mg. Initially, crospovidone and mannitol were forced through a wire sieve with the aperture size of 425 µm before blending with DPH or optimized microparticles (equivalent to 5 mg of DPH), spray-dried lactose monohydrate, and microcrystalline cellulose with geometric mixing technique. Then, PVP K-30 and magnesium stearate was separately added and blended for 1 min. The solid mixtures were weighed and compressed by a hydraulic press machine (SPECAC15011, Specac Ltd., Orpington, UK) using a 9.65-mm diameter flat-surface punch with a compression force of 1 ton and dwelling time of 10 s. The tablets were kept in air-tight plastic bags and stored in a desiccator for further evaluation.

### 2.7. Characterization of ODTs

#### 2.7.1. Thickness, and Hardness Measurement

The hardness tester (TBH225TD, Erweka GmbH, Langen, Germany) was used to determine the thickness, and hardness of 20 tablets for each formulation. 

#### 2.7.2. Friability Test

The USP compendial approach was used to conduct the friability test with the friability tester (TA120, Erweka GmbH, Germany). A total of 6.5 g of ODTs were collected. Before placing the tablets in the friability drum, any dust was removed and the drum was rotated at 25 rpm for 4 min. Following test completion, the samples were dedusted and accurately weighed. The friability was calculated using the following formula:

(6) $$\mathrm{Friability}=\frac{\mathrm{Initial}\;\mathrm{weight}\;\left(g\right)\;-\;\mathrm{Final}\;\mathrm{weight}\;(g)}{\mathrm{Initial}\;\mathrm{weight}\;(g)}\;\times \;100$$

#### 2.7.3. In Vitro Disintegration Test

The in vitro disintegration test was conducted based on the research work of Hoashi and coworkers [18]. The apparatus setup comprises of two meshes, a 20-g ring weight, and pipette filled with disintegration test medium (SSF, pH 6.75). Initially, an ODT was carefully placed on the center of lower mesh and covered by upper mesh, with a 20-g ring weight on top. Then, SSF was dropped on the tablet with the flow rate of 4 mL/min. An in vitro disintegration time was calculated by measuring the time required for the upper mesh to completely contact the lower mesh using video capturing. All experiments were performed in triplicate.

#### 2.7.4. In Vitro Dissolution Test 

The in vitro dissolution test of ODTs was performed in triplicate using the above-mentioned method described in Section 2.5.8. However, a 12-mm inner diameter tablet cell was used instead of cell for powders and granulates. 

#### 2.7.5. In Vivo Evaluation

The in vivo evaluation of DPH-ODTs and OM-ODTs was performed in 6 healthy volunteers (2 men and 4 women, ages 23 to 30), according to a research protocol approved by the Ethics Committee for Human Research of Silpakorn University (COE 63.0922-079, approved on the 22 September 2020). 

##### Perception and Bitterness Threshold Determination

Briefly, the perception and bitterness recognition threshold of DPH was first determined. The series of DPH solution were prepared with deionized water: (A) 0 µg/mL, (B) 12.5 µg/mL, (C) 25 µg/mL, (D) 37.5 µg/mL, (E) 50 µg/mL, (F) 62.5 µg/mL, (G) 75 µg/mL, (H) 87.5 µg/mL, and (I) 100 µg/mL. Participants were instructed to rinse their mouth with deionized water before tasting 10 mL of solution A, and hold it in their mouth for 30 s. After that, they were asked to try solution E, which had a moderate concentration. They were asked to rate the bitterness by choosing one of the following perceptions after fully rinsing their mouths with water.

1. I did not feel any difference between solution A and E
2. I felt something, but I cannot differentiate the taste
3. I felt a bitter taste

The subjects who answered 1 or 2 were then instructed to try solution F, which had a higher concentration. In the meantime, those who answered 3 were instructed to try solution D, which had a lower concentration. The wash-out duration between each solution test was 10 min.

##### Palatability Test

A participant training session was held to guarantee that the results were comparable. They were given the opportunity to sample a variety of solutions containing varying levels of DPH. After that, each solution’s bitterness rating (0 to 100) was revealed.

**Figure 1:**
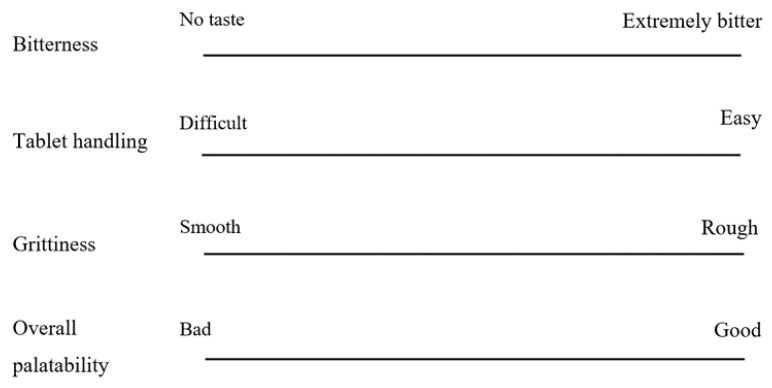
VAS line of bitterness, tablet handling, grittiness, and overall palatability.

The palatability test of DPH-ODTs or OM-ODTs was then conducted at random. The participants were instructed to rinse their mouth with deionized water before tasting a tablet that was placed in the center of their tongue. A stopwatch was used to time the disintegration of each tablet. The participants were instructed to hold the tablet for another 30 s after it had totally disintegrated. They were, however, allowed to spit out the sample if the taste was too strong. After finishing, they were asked to rate the sample on bitterness, tablet handling, grittiness, and overall palatability using a 100-mm visual analog scale (VAS) line (Figure 1). The marks were measured in millimeters, and the median VAS score of DPH-ODTs and OM-ODTs were compared using a paired-samples T-test.

## 3. Results and Discussion 

### 3.1. Study on Factors Affecting Microparticle Preparation

**Figure 2:**
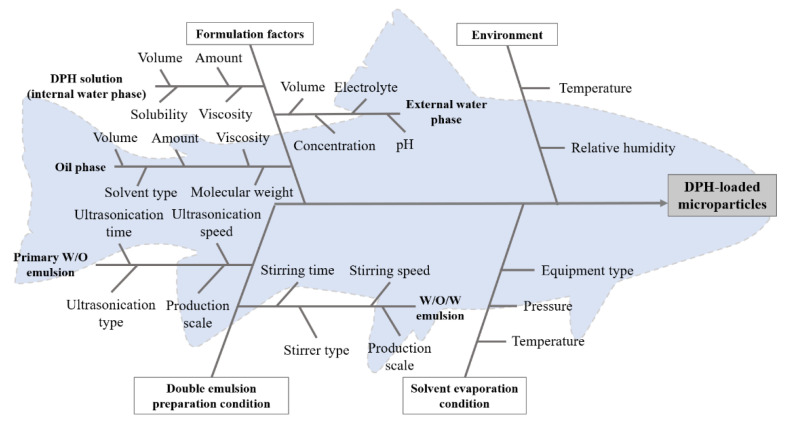
Fishbone (Ishikawa) diagram of factors affecting microparticle preparation.

Based on information from the literature on highly water-soluble and small molecules encapsulating using DESE [9,10,11,12,19,20,21,22,23,24,25], the factors affecting microparticle preparation (i.e., materials, environment, double emulsion preparation condition, and solvent evaporation condition) were determined, as illustrated in the fishbone (Ishikawa) diagram in Figure 2.

Throughout the experiment, the material used, ultrasonicator employed in the first emulsification, stirrer used in the second emulsification, solvent evaporation condition, and production scale were all kept constant, while temperature and relative humidity were monitored. As a result, these variables were left out of the risk assessment. The risk matrix (Table 2) was then created based on the published data in order to prioritize the effect of these factors on critical quality attributes (CQAs) of DPH-loaded microparticles. Particle size and drug dissolution were the CQAs of DPH-loaded microparticles for bitter taste masking and ODT production.

The studies were then carried out to investigate the impact of high-risk parameters on particle size and drug dissolution, such as polymer amount, stabilizer concentration, volume of external water phase, and stirring time in the second emulsification.

### 3.2. Experimental Design 

According to the literature and preliminary research, independent factors such as the AMC amount (X_1_), stirring duration (X_2_), and W_2_ volume altered the characteristics of microparticles (X_3_) which in turn affected the characteristics of microparticles. During the experiment, the environmental factors including temperature and relative humidity were monitored. The evaluated responses were particle size (Y_1_), cumulative in vitro drug dissolution at 5 min in SSF (Q_5_; Y_2_), and MDT in SGF (Y_3_). Based on the preliminary investigation, the levels of independent factors were chosen (Table 3). The results of BBD with the total 17 experimental runs and central points are also shown in Table 3.

#### 3.2.1. Effect of Independent Factors on Particle Size

The effect of independent variables on the particle size of DPH-loaded microparticles was investigated. It was found that the particle size ranged from 82.50 to 247.73 µm (Table 3). The relationship between independent factors and particle size was described in terms of coded factors using Equation (7). The ANOVA results (Table 4) demonstrated a *p*-value less than 0.05, indicating that the model was reliable. The suitability of the model was represented by lack-of-fit with *p*-value of 0.0954 and the goodness of fit was represented by an R^2^ value of 0.9820.

Particle size = 183.08 + 62.94 X_1_ − 14.59 X_2_ − 17.17 X_1_^2^ (7)

**Figure 3:**
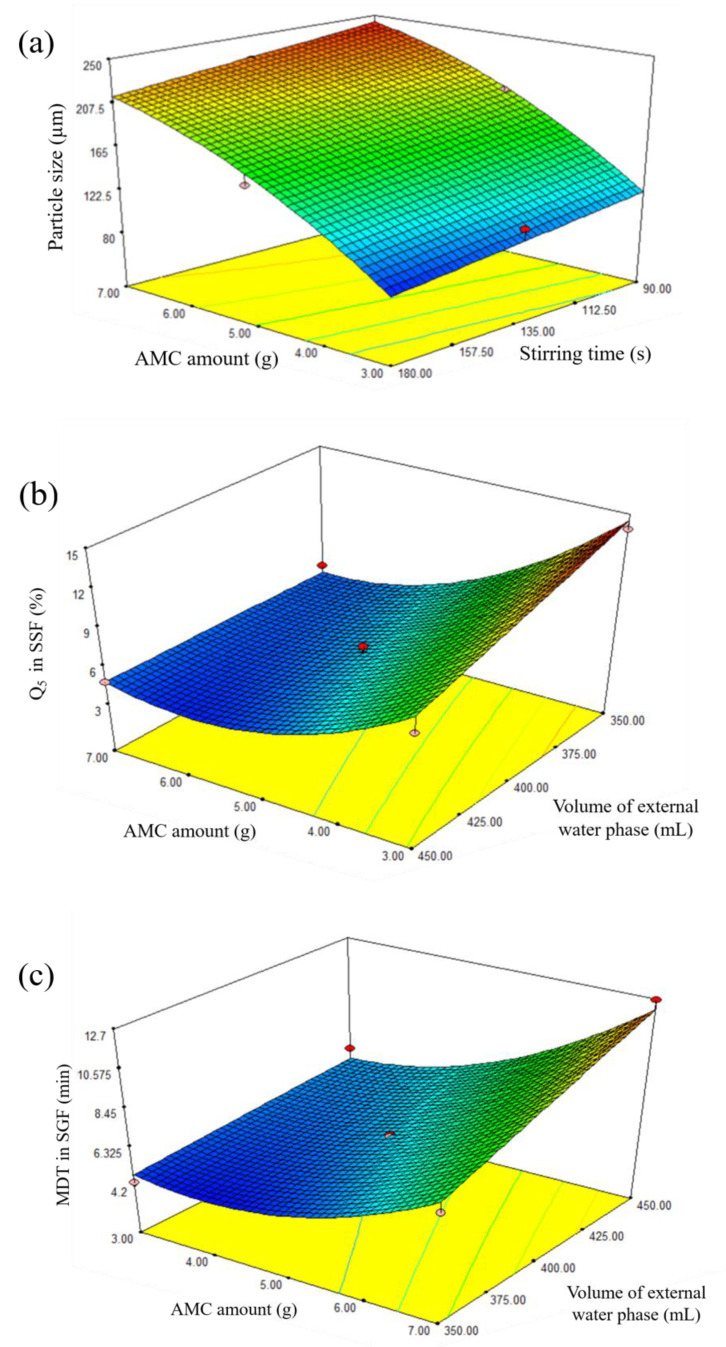
Three-dimensional plots showing the effect of independent factors on (**a**) size of microparticles, (**b**) Q_5_ in SSF, and (**c**) MDT in SGF.

The results showed that X_1_, X_2_, and X_1_^2^ were significant terms, meaning that AMC amount and stirring time significantly influenced the particle size. The influence of these factors is shown in a three-dimensional plot (Figure 3a), which shows that particle size increased as AMC quantity and stirring time decreased. The results were in good agreement with previous study [16,17]. The viscosity of the oil phase may explain these events. Because the viscosity of the oil phase increased as the AMC quantity increased, more stirring force and time were required to disperse the primary emulsion in the external water phase [8,12]. In order to avoid the internal emulsion droplets break up, it is advised that a low shear force can be employed to disseminate the primary W_1_/O emulsion in the external water phase during the second emulsification step [26]. In this study, the stirring rate was kept constant at 500 rpm while the stirring time was varied. The model indicated a negative relationship between stirring time and microparticle size, which might be attributed to the longer emulsification interval caused by increased stirring duration. As a result, the particle size was decreased, as reported in a prior publication [27].

#### 3.2.2. Effect of Independent Factors on Q_5_ in SSF

According to the data, the lowest percentage was 3.64% and the highest was 14.23% in Q_5_. The model predicting Q_5_ of DPH-loaded microparticles was significant with a *p*-value of less than 0.05, according to the ANOVA results (Table 5). The model adequacy was shown by a *p*-value of 0.1384 for lack-of-fit, and the R^2^ value of 0.9216 for goodness of fit. In Equation (8), a reduced mathematical model was represented in coded terms, suggesting that the most critical factor was the amount of AMC, and that both the amount of AMC and the volume of external water phase had a negative relationship in the model.

Q_5_ in SSF = 5.72 − 3.61 X_1_ − 1.24 X_3_ + 1.29 X_1_ X_3_ + 2.68 X_1_^2^ (8)

**Figure 4:**
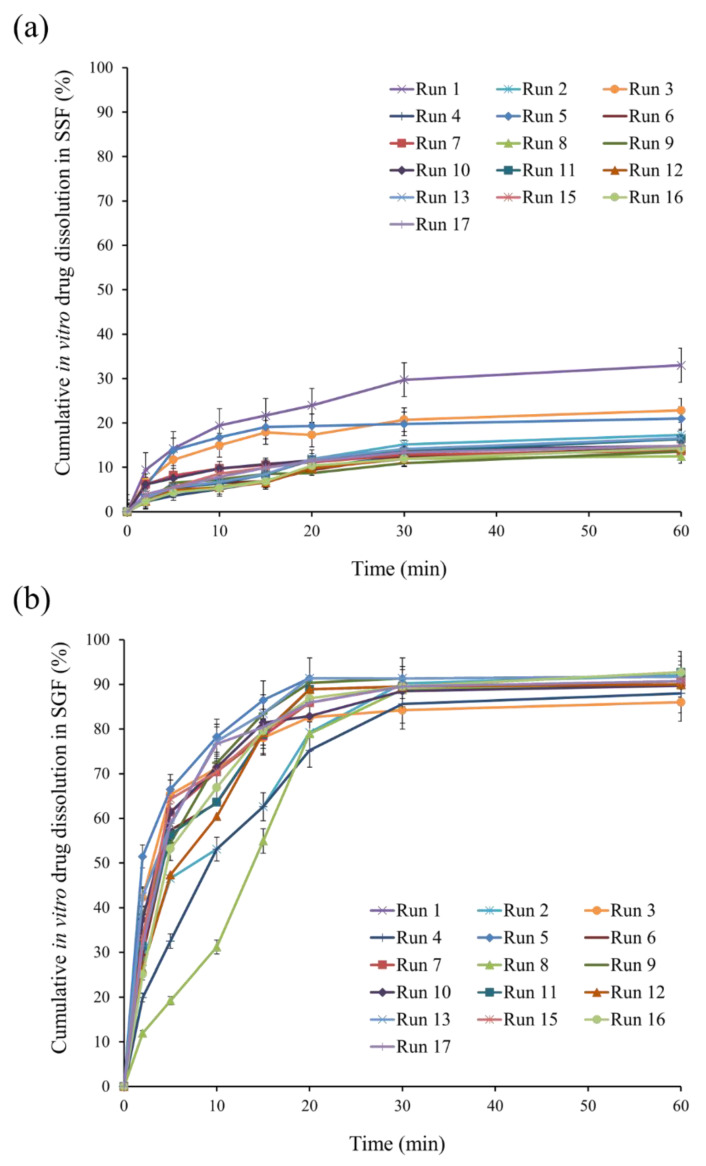
In vitro drug dissolution profiles of standard runs in (**a**) SSF and (**b**) SGF.

The three-dimensional plot (Figure 3b) shows the effect of AMC amount and volume of external water phase on Q_5_ in SSF. In vitro drug dissolution profiles in SSF of standard runs are shown in Figure 4a. For instance, Q_5_ for runs 5, 7, and 8 was 13.90%, 8.21%, and 4.79%, respectively, indicating delayed drug dissolution behavior. These phenomena can be explained by AMC properties, a pH-dependent solubility polymer that is soluble at pH less than 5, yet swellable and permeable above pH 5. An increase in AMC amount from 3 g (run 7) to 7 g (run 8) may result in the formation of a thicker swellable polymer layer in SSF, preventing DPH dissolution from microparticles and resulting in a lower Q_5_. In the model, the volume of the exterior water phase had a negative association. In vitro drug dissolution profiles from SSF runs 5 and 7 revealed that increasing the volume of external water phase from 350 mL (run 5) to 450 mL (run 7) resulted in a lower Q_5_. This can be explained by the role of the external water phase, which is responsible for the final step of solvent evaporation. The steps of microparticle hardening were briefly described as follows: organic solvent diffusion within emulsion droplets, organic solvent diffusion to the intermediate boundary between dispersed emulsion droplet and external phase, and organic solvent evaporation to ambient environment [8]. Due to the higher volume available for organic solvent removal, an increase in the amount of external water phase resulted in rapid organic solvent diffusion, improving microparticle hardness [28,29]. As a result, it is hypothesized that this phenomenon was relevant to the formation of complex microparticle structures, which resulted in a low Q_5_.

#### 3.2.3. Effect of Independent Factors on MDT in SGF

Ideally, the taste-masking technique should only mask the unpleasant taste of the drug without altering its biopharmaceutical behavior [18,30]. As a result, the effect of independent factors on MDT in SGF was studied. The result showed that MDT ranged from 4.34 min in run 5 to 12.67 min in run 8. The results of the ANOVA test (Table 6) revealed that a reduced mathematical model predicting MDT of DPH-loaded microparticles was statistically significant with a *p*-value of less than 0.05 and a *p*-value for lack-of-fit of 0.1104, confirming model suitability. Furthermore, the R^2^ value of 0.9379 indicated the goodness of fit. Equation (9) represented the model in coded terms. The three-dimensional plot in Figure 3c illustrates the effect of AMC quantity and volume of external water phase on MDT.

MDT in SGF = 6.14 + 2.44 X_1_ + 1.24 X_3_ + 0.78 X_1_ X_3_ + 1.57 X_1_^2^ (9)

The in vitro drug dissolution profiles from runs 5, 7, and 8 in SGF are shown in Figure 4b, demonstrating that increasing the AMC amount and volume of W_2_ resulted in an increase in MDT. This phenomenon can be described similarly to Q_5_ in SSF. As a result of the increased AMC amount, a thicker drug-dissolving barrier resulted in a higher MDT. Increased W_2_ volume resulted in rapid emulsion hardening, which strengthened microparticle structure [12,31], resulting in an increase in MDT.

#### 3.2.4. Validation of Mathematical Model

To validate the correlation among the actual and predicted value of the proposed mathematical model, an additional experiment was performed (Table 7). The results demonstrated RMSE of particle size, Q_5_, and MDT of 11.63, 0.50, and 1.06, respectively, indicating the model’s effectiveness. 

#### 3.2.5. Optimization 

After validation of the model, a good predictability of the model was obtained. The optimization was carried out to find an optimized formulation to prepare desired taste-masked DPH-loaded microparticles containing 17.5% DPH on average. Since we set out to make ODTs that disintegrate in the mouth, the disintegrated particles could generate unpleasant or grittiness on the tongue, leading to poor patient compliance. Recent research have shown a link between particle size and grittiness; the smaller the particle size, the lesser the grittiness [32,33]. These results revealed that the particle size of greater than 200 µm may cause intense grittiness feeling. Additionally, small particle size results in high surface energy, leading to the formation of agglomerates. Therefore, a target particle size range of 100 to 200 µm was determined.

Taste-masking is deemed successful when drug dissolution in the first 5 min is less than 10%, according to the FIP/AAPS guidelines for dissolution/in vitro dissolution testing of novel/special dosage forms [29]. In this study, drug dissolution testing in 50 mL of SSF was carried out to evaluate drug-loaded microparticles equivalent to 5 mg DPH. Based on the guideline, the taste-masking is achieved when drug dissolution is less than 0.5 mg (or 10 µg/mL). However, the bitterness threshold of the drug in question is also necessary to take into consideration for developing decision-making criteria. According to the result of in vivo taste evaluation study in human volunteers, the bitterness threshold of DPH was 56.3 ± 15.73 µg/mL, indicating that taste-masking was achieved when the amount of drug dissolution amount was below this concentration. Compared to that of FIP/AAPS guidelines [29], the evaluation of taste-masking using drug dissolution of less than 10% at initial 5 min was chosen as a strict interpretation for taste-masking evaluation.

As mentioned in the previous section, the ideal taste-masking approach should not affect the biopharmaceutical properties of native drugs. Owing to the high solubility and high permeability properties of DPH, drug dissolution in 0.1 N HCl of over 85% within 15 min is acceptable. From this study, the in vitro dissolution of DPH in SGF showed a rapid drug dissolution (more than 85% within 10 min) with MDT of 4.35 min. The MDT threshold of less than 10 min was found to be acceptable after considering the aforementioned suggestion and our findings.

In brief, the optimization criteria for determining the optimum values used to prepare DPH-loaded microparticles include particle sizes ranging from 100 to 200 µm, Q_5_ less than 10%, and MDT less than 10 min. Subsequently, the optimal value of AMC amount of 5.7 g, stirring time of 179 s, and volume of W_2_ of 350 mL were obtained. These values predicted microparticle size of 189.5 µm, Q_5_ of 5.5%, and MDT of 5.7 min with desirability of 0.73. The predicted values were validated by further measuring particle size, in vitro drug dissolution both in SSF and SGF. The optimized microparticles showed particle size of 174.45 ± 18.19 µm, Q_5_ of 5.04%, and MDT of 5.97 min. Comparing to the predicted value, percentage error of particle size, Q_5_, and MDT were 14.65%, 9.13%, and 4.52%, confirming the validity of the model.

### 3.3. Characterization of Optimized Microparticles

**Figure 5:**
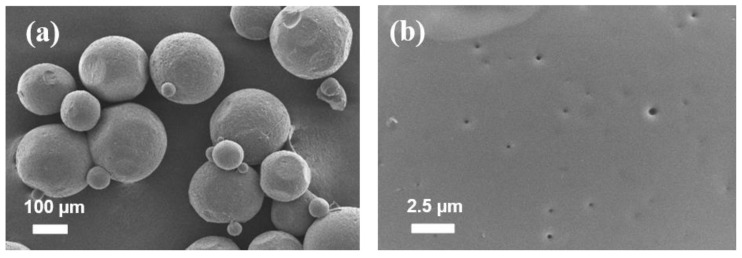
SEM images of microparticles and surface morphology of optimized microparticles; (**a**) particles and (**b**) surface morphology. Scale bars are shown on individual photographs.

The optimized microparticles prepared with dichloromethane produced regular, highly spherical microparticles with a smooth surface, as seen in Figure 5. Because of the inherent toxicity of organic solvent [34], a residual solvent test was required to ensure that the remaining dichloromethane met the limiting criteria, which includes a concentration limit of 600 ppm. The quantity of residual dichloromethane detected in microparticles was less than 5 ppb, according to the results. 

**Figure 6:**
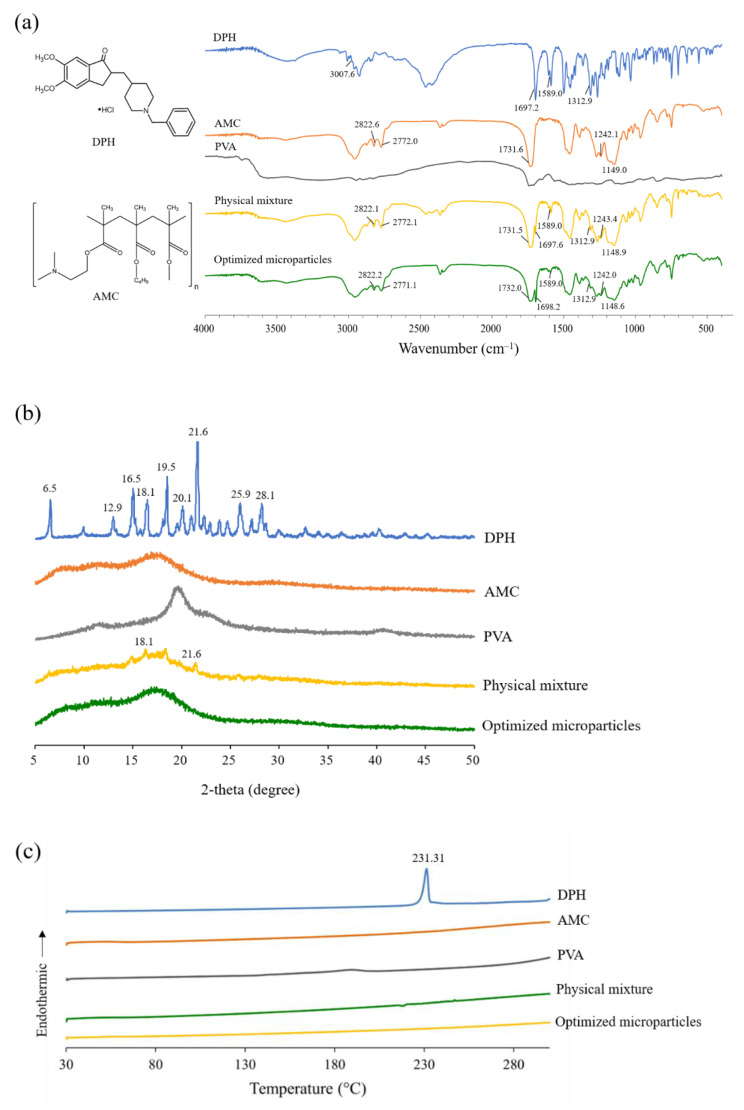
(**a**) FTIR spectra, (**b**) PXRD patterns, and (**c**) DSC thermograms of DPH, AMC, PVA, physical mixture, and optimized microparticles.

FTIR was used to investigate the chemical compatibility of DPH, AMC, PVA, their physical mixture, and optimized microparticles. DPH showed a band at 3007.6 cm^−1^, which was attributed to an aromatic C-H stretching, as illustrated in Figure 6a. A band at 1697.2 cm^−1^ was observed due to the stretching of the C=O group on the indanone moiety, while a band at 1589.0 cm^−1^ was assigned to the vibration of aromatic C=C. Due to the C-N stretching on the piperidine ring, DPH also revealed a band at 1312.9 cm^−1^. In case of AMC, a band on the AMC spectrum was observed at 2822.6 and 2772.0 cm^−1^ due to the C-H stretching of the dimethyl amino group. There was also a strong C-O stretching band at 1149.0 and 1242.1 cm^−1^, as well as a C=O band at 1731.6 cm^−1^, suggesting the presence of the ester group. The superimposed spectrum between DPH and AMC was clearly observed in the case of physical mixture. Similar findings were also observed in the spectrum of optimized microparticles. After preparation, there was no notable deviation in the characteristic band of optimized microparticles, indicating compatibility of components.

The sharp diffraction peaks were seen at 2-theta values of 6.5, 12.9, 16.5, 18.1, 19.5, 20.1, 21.6, 25.9, and 28.1 degrees in the PXRD pattern of DPH shown in Figure 6b, suggesting its crystalline structure. Similarly, the sharp peaks were also observed in the PXRD pattern of physical mixture at 2-theta values of 18.1 and 21.6, which were attributed to the crystalline peak of DPH. In the case of AMC, PVA, and optimized microparticles, however, a halo pattern was found, indicating that these substances are amorphous. The absence of a crystalline peak in the PXRD pattern of optimized microparticles further demonstrated that they were completely encapsulated.

To investigate the interaction between the components, DSC thermograms of DPH, AMC, PVA, their physical mixture, and optimized microparticles were investigated (Figure 6c). A prominent endothermic peak at 231.3 °C was observed in the DSC thermogram of DPH, which was attributed to the melting peak. A small endothermic peak was observed on the thermogram of the physical mixture. The absence of an endothermic peak in the thermogram of optimized microparticles, as a result, confirmed the PXRD findings. It can be hypothesized that the crystalline state of DPH was transformed to an amorphous state, and it was completely incorporated in the microparticles.

### 3.4. Preparation and Evaluation of ODTs

#### 3.4.1. Physical Properties of ODTs

The 200-mg ODTs were prepared by direct compression with the addition of DPH or DPH-loaded optimized microparticles. The obtained ODTs were found to be off-white in color, round, and flat-faced. The DPH-ODTs had a smooth surface, but OM-ODTs had a rough surface due to the dispersion of optimized microparticles. Table 8 shows the physical properties of DPH-ODTs and OM-ODTs. The tablet thickness of DPH-ODTs was almost similar to that of OM-ODTs. The tablet hardness of DPH-ODTs was roughly 4 N lower than that of OM-ODTs.

#### 3.4.2. In Vitro Evaluation of ODTs

**Figure 7:**
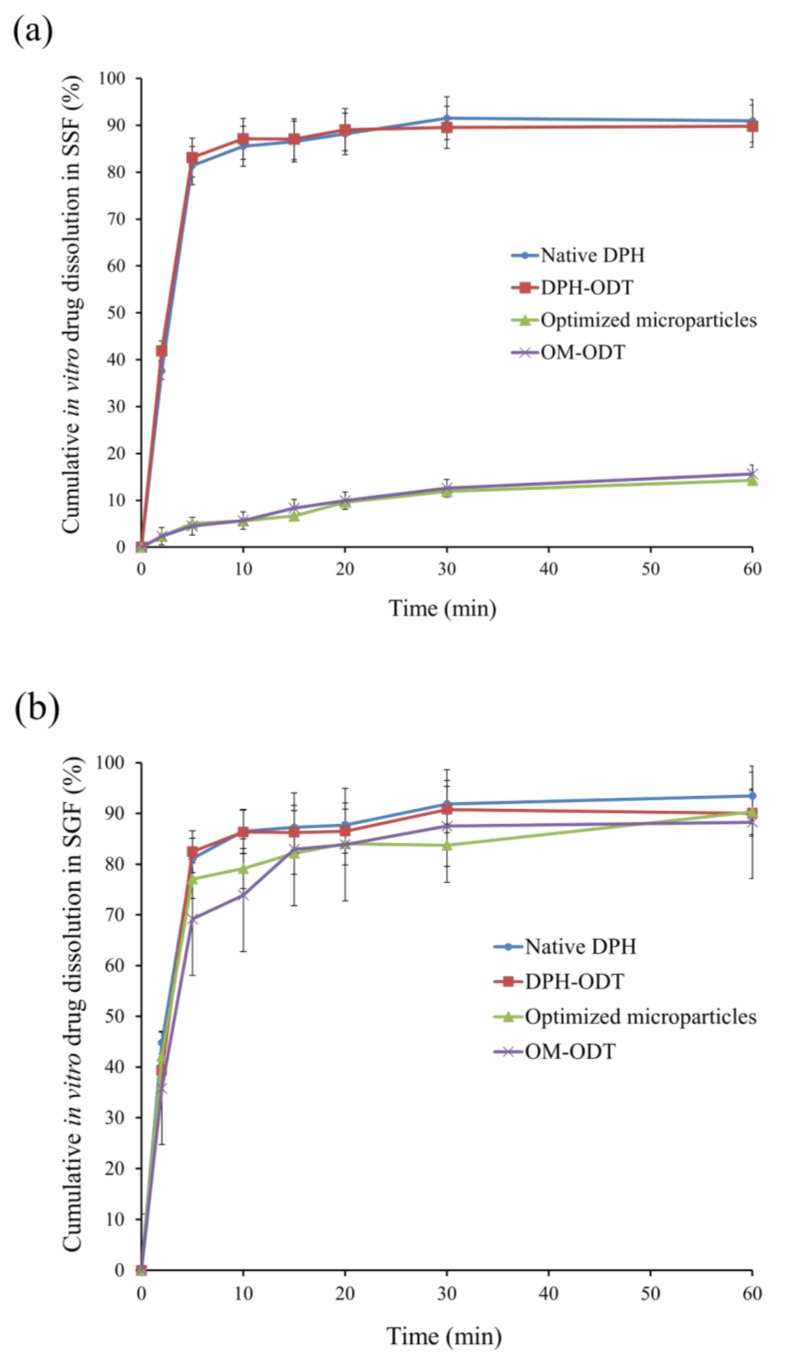
In vitro drug dissolution profiles of the samples in (**a**) SSF, and (**b**) SGF.

For the ODTs, the tablets should rapidly disintegrate with disintegration time of 30 s or less. No compendial disintegration test was specifically designed for ODTs in the pharmacopeia. There are two stages involved in the disintegration of ODT when considering physiological conditions: saliva is absorbed once the tablet is placed on the tongue, followed by tablet fragmentation induced by pressure between the tongue and the hard palate. To simulate these steps, in vitro disintegration of all ODTs was determined according to the method proposed by Hoashi and coworkers [18]. As shown in Table 8, the in vitro disintegration time of DPH-ODTs and OM-ODTs was 13.0 ± 0.8, and 14.0 ± 1.2, respectively. Friability results showed that both DPH-ODTs and OM-ODTs had less than 1% friability. The lower friability of OM-ODTs may be attributed to its higher hardness. The in vitro dissolution of DPH, DPH-ODTs, optimized microparticles, and OM-ODTs were determined in both SSF and SGF (Figure 7). 

The dissolution profiles of DPH and DPH-ODTs in SSF, as shown in Figure 7a, demonstrated rapid drug dissolution of more than 80% in 5 min, with Q_5_ values of 81.38% and 83.11%, respectively. Optimized microparticles and OM-ODTs, on the other hand, demonstrated delayed dissolution behavior with Q_5_ of 5.04% and 4.48%, respectively. In addition, drug dissolution in SGF (pH 1.2) was also performed to determine drug dissolution in simulated gastric condition [35]. The rapid drug dissolution of DPH and DPH-ODTs of over 80% within 5 min was observed (Figure 7b). The dissolution of optimized microparticles and OM-ODTs was delayed and completed within 15 min, with MDTs of 5.95 min and 5.01 min, respectively. The optimized microparticles containing AMC suppressed drug dissolution at pH 6.75 in SSF but allowed the drug to dissolve at gastric pH, according to the findings. The taste-masking ability of the optimized microparticles and OM-ODTs has been confirmed, as Q_5_ was less than 10%.

#### 3.4.3. In Vivo Disintegration Test and Taste Evaluation of ODTs

Initially, the perception and bitterness threshold of DPH was determined. The perception threshold was found to be 41.7 μg/mL (ranged from 25.0 to 62.5 μg/mL, SD of 13.8). While the bitterness threshold of DPH was 56.3 μg/mL (ranged from 37.5 to 75.0 μg/mL, SD of 15.7), which is consistent with previous reports [36,37].

**Figure 8:**
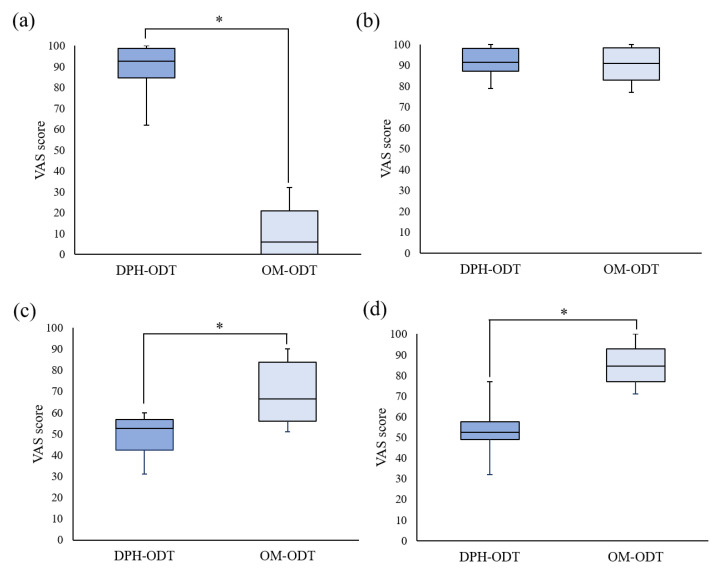
The box-whisker plot of VAS score of (**a**) bitterness score, (**b**) tablet handling, (**c**) grittiness, and (**d**) overall palatability. Note: The statistically significant difference (*p* < 0.05) between DPH-ODTs and OM-ODTs is noted by an asterisk.

The average disintegration time of DPH-ODTs and OM-ODTs, as determined by the participants, was found to be 22.6 ± 3.4 and 18.8 ± 1.7 s, respectively, which were considerably different from the in vitro disintegration time. Huanbutta et al. [38,39] also found that the disintegration time in the human oral cavity is substantially shorter than in vitro conditions. To assess the palatability, the participants were asked to hold the ODTs in their mouth for 30 s after they had completely disintegrated, and to draw a line on a VAS line. In Figure 8, the VAS score of ODTs was represented as a median with a range in a box-whisker plot, with an asterisk indicating a statistically significant difference between two samples at a *p*-value less than 0.05. The median VAS bitterness score, as shown in Figure 8a, of OM-ODTs (92.5) was significantly lower than that of DPH-ODTs (6), showing that DPH bitterness was efficiently suppressed. In contrast, the median VAS tablet handling of DPH-ODTs and OM-ODTs (Figure 8b) was comparable, indicating that tablet handling was not significantly different between these formulations. The median grittiness score of OM-ODTs was significantly higher than that of DPH-ODTs, indicating that the grittiness feeling of OM-ODTs was more prominent than that of DPH-ODTs, as shown in Figure 8c. These results can be explained by the large particle size of optimized microparticles, whereas DPH is a fine powder. However, the median VAS score of overall palatability of OM-ODTs was significantly higher than that of DPH-ODTs (Figure 8d), indicating that OM-ODTs was acceptable. In summary, the OM-ODTs were found to have a pleasant taste with a good mouth feeling that had some grittiness and rapidly disintegrated in the oral cavity.

## 4. Conclusions

The bitter taste of DPH is the primary problem in the development of ODTs, therefore, taste-masking is required. The taste-masked microparticles containing DPH with the particle size of 174.45 ± 18.19 µm, Q_5_ of 5.04%, and MDT of 5.97 min were successfully prepared using optimized conditions obtained from BBD including AMC amount of 5.7 g, stirring time of 179 s, and volume of W_2_ of 350 mL. The optimized microparticles were further formulated to taste-masked ODTs using the direct compression method and evaluated in vitro and in vivo. The in vitro dissolution results of optimized microparticles and OM-ODTs in SSF showed the delayed dissolution pattern with Q_5_ of less than 10%, indicating that taste-masking was achieved. In contrast, in gastric conditions, these formulations reached over 80% drug dissolution within 5 min, indicating an immediate release in the stomach. The in vivo evaluation revealed that the ODTs with taste microparticles had a pleasant taste, were easy to handle, and had some grittiness. Overall palatability, on the other hand, was thought to have improved. The in vivo disintegration test revealed that the ODTs disintegrated quickly in the oral cavity of the human subjects, with a disintegration time of less than 30 s. As a conclusion, our findings highlight the benefits of using quality risk assessment and design of experiment techniques for dosage form design, as well as the accomplishment of taste-masking.

## Figures and Tables

**Table 1:** Formulation of ODT containing DPH and optimized microparticles.

Ingredients	Quantity (mg/tablet)	
	DPH-ODT	OM-ODT
DPH	5	-
Optimized microparticles	-	28.5
Mannitol	90	90
Spray-dried lactose monohydrate	72	48.5
Microcrystalline cellulose	21	21
Crospovidone	8	8
PVP K-30	2	2
Magnesium stearate	2	2

**Table 2:** Initial risk assessment for the formulation factors and operating conditions of microparticle preparation by DESE.

Factors	CQAs	
	Particle Size	Drug Dissolution
Formulation factors:		
Drug amount	Medium	Low
Organic solvent amount	Low	Low
Polymer amount	High	High
Stabilizer concentration	High	High
pH of external water phase	Low	Low
Volume of internal phase	Medium	Medium
Volume of oil phase	Medium	Low
Volume of external water phase	High	High
Operating conditions:		
Ultrasonication time in the first emulsification	Medium	Low
Stirring time in the second emulsification	High	High
Stirring rate in the second emulsification	Medium	Low

**Table 3:** Independent factors, level of each factor, and responses in the Box-Behnken design of DPH-loaded microparticles preparation.

**Parameter**				**Level**		
				**Low (−1)**	**Medium (0)**	**High (+1)**
Independent factors						
X_1_: AMC amount (g)				3	5	7
X2: Stirring time (s)				90	135	180
X3: Volume of outer water phase (mL)				350	400	450
Responses						
Y1: Particle size (µm)						
Y2: Q5 in SSF (%)						
Y3: MDT in SGF (min)						
**Standard Run Order**	**Independent Factors**			**Experimental Values of Responses**		
	**X_1_**	**X_2_**	**X_3_**	**Y_1_**	**Y_2_**	**Y_3_**
1	3	90	400	116.24	14.23	5.15
2	7	90	400	247.73	5.41	9.86
3	3	180	400	82.50	11.71	5.27
4	7	180	400	213.02	3.64	10.52
5	3	135	350	113.82	13.90	4.34
6	7	135	350	226.01	5.32	7.56
7	3	135	450	99.31	8.21	6.32
8	7	135	450	228.61	4.79	12.67
9	5	90	350	194.25	6.50	5.78
10	5	180	350	157.52	7.57	6.03
11	5	90	450	186.28	5.45	7.54
12	5	180	450	174.72	4.93	7.11
13	5	135	400	184.82	5.46	5.17
14	5	135	400	187.53	6.20	5.67
15	5	135	400	193.62	5.54	6.14
16	5	135	400	185.23	4.35	7.78
17	5	135	400	183.72	5.47	6.03

**Table 4:** ANOVA results of the fitted model for predicting Y_1_.

Source	Sum of Squares	Degree of Freedom	Mean Square	F-Value	*p*-Value, prob > F
Y_1_					
Model	34,641.40	3	11,547.13	236.38	<0.0001
X_1_	31,689.03	1	31,689.03	648.72	<0.0001
X_2_	1703.53	1	1703.53	34.87	<0.0001
X_1_^2^	1248.84	1	1248.84	25.57	0.0002
Residual	635.04	13	48.85		
Lack of fit	572.29	9	63.59	4.05	0.0954
Pure error	62.75	4	15.69		
Cor total	35,276.44	16			
Regression coefficient: R^2^ = 0.9820, adjusted R^2^ = 0.9778, predicted R^2^ = 0.9693					

**Table 5:** ANOVA results of the fitted model for predicting Y_2_.

Source	Sum of Squares	Degree of Freedom	Mean Square	F-Value	*p*-Value, prob > F
Y_2_					
Model	153.73	4	38.43	35.25	<0.0001
X_1_	104.33	1	104.33	95.69	<0.0001
X_3_	12.28	1	12.28	11.26	0.0057
X_1_X_3_	6.66	1	6.66	6.11	0.0294
X_1_^2^	30.47	1	30.47	27.95	0.0002
Residual	13.08	12	1.09		
Lack of fit	11.31	8	1.41	3.19	0.1384
Pure error	1.77	4	0.44		
Cor total	166.82	16			
Regression coefficient: R^2^ = 0.9216, adjusted R^2^ = 0.8954, predicted R^2^ = 0.7923					

**Table 6:** ANOVA results of the fitted model for predicting Y_3_.

Source	Sum of Squares	Degree of Freedom	Mean Square	F-Value	*p*-Value, prob > F
Y_3_					
Model	72.92	4	18.23	45.27	<0.0001
X_1_	47.68	1	47.68	118.39	<0.0001
X_3_	12.33	1	12.33	30.61	0.0001
X_1_X_3_	2.45	1	2.45	6.08	0.0297
X_1_^2^	10.47	1	10.47	26.00	0.0003
Residual	4.83	12	0.40		
Lack of fit	4.26	8	0.53	3.71	0.1104
Pure error	0.57	4	0.14		
Cor total	77.76	16			
Regression coefficient: R^2^ = 0.9379, adjusted R^2^ = 0.9171, predicted R^2^ = 0.8217					

**Table 7:** Comparison of the difference between actual and predicted value of responses for model validation.

**Independent Factors**			
**No.**	**AMC Amount (g)**	**Stirring Time (s)**	**Volume of Water Phase (mL)**
F1	5.5	120	350
F2	4.8	160	380
F3	5.2	100	430
**Responses**			
**No.**	**Actual value**	**Predicted value**	**RMSE**
Particle size (µm)			
F1	209.29	202.60	11.63
F2	159.20	168.50	
F3	217.11	200.55	
Q_5_ in SSF (%)			
F1	5.51	5.90	0.50
F2	6.97	6.65	
F3	5.43	4.72	
MDT in SGF (min)			
F1	6.49	5.41	1.06
F2	6.62	5.45	
F3	8.10	7.19	

**Table 8:** Physical properties of ODTs.

Formulation	Thickness (mm) ± SD	Hardness (N) ± SD	In Vitro Disintegration Time (s) ± SD	Friability (%)	In Vivo Disintegration Time (s) ± SD
DPH-ODTs	2.18 ± 0.02	38.0 ± 2.8	13.0 ± 0.8	0.90	22.6 ± 3.4
OM-ODTs	2.34 ± 0.05	42.0 ± 0.8	14.0 ± 1.2	0.76	18.8 ± 1.7

## Data Availability

Data is contained within the article.
